# Combinational Effects of Prebiotics and Soybean against Azoxymethane-Induced Colon Cancer * In Vivo*

**DOI:** 10.1155/2011/868197

**Published:** 2011-09-26

**Authors:** V. P. Gourineni, M. Verghese, J. Boateng, L. Shackelford, N. K. Bhat, L. T. Walker

**Affiliations:** ^1^Nutrition Biochemistry and Carcinogenesis Laboratory, Department of Food and Animal Sciences, Alabama A&M University, Normal, AL 35762, USA; ^2^Department of Food Science, Alabama A&M University, P.O. Box 1628, Normal, AL 35762, USA; ^3^Department of Chemistry, Alabama A&M University, Normal, AL 35762, USA

## Abstract

Prebiotic fructans are nondigestible carbohydrates with numerous health benefits. Soybean is a rich source of phytonutrients such as isoflavones. The objective of this study was to evaluate the chemopreventive effects of prebiotics (Synergy1) and soybean meal (SM) at 5% and 10% levels alone and in combination on azoxymethane- (AOM-) induced colon carcinogenesis. After one wk of acclimatization, Fisher 344 male rats (*N* = 90) were randomly assigned to 9 groups (*n* = 10). Control rats (C) were fed AIN-93G/M. Two s/c injections of AOM were administered to rats at 7 and 8 wk of age at 16 mg/kg body weight. Rats were killed by CO_2_ asphyxiation at 45 wk. Tumor incidence (%) in treatment groups ranged from 40 to 75 compared to 100 in C. Results indicate that feeding prebiotics and soybean in combination significantly reduced incidence of AOM-induced colon tumors with implications for food industry in the food-product development.

## 1. Introduction

Cancer is the second most common cause of deaths after heart disease and accounts for one of every four deaths in the US [1]. Despite advances in technology and public health awareness, colon cancer prevalence is expected to increase in aged population adding economic burden to the nation [2].

Gut-associated cancers are influenced by diet [3]. Epidemiological and experimental studies showed relation between dietary consumption patterns and prevention of chronic diseases [4, 5]. Research on diet-disease correlation using epidemiological and animal experiments showed single nutrient effects in disease prevention [6–8]. However, nutrition-health interface becomes more apparent by exploring the synergistic action of foods in animal models [9]. Recently, research is focused on identifying specific combinations of phytochemicals or foods offering greater chemopreventive potential. Understanding the influence of various bioactive compounds on molecular interactions and immunomodulatory responses led to the emerging strategy of combinational chemoprevention [10].

Prebiotics are associated positively in the prevention of colon cancer by modulating colonic environment [11]. A combination of long-chain inulin and short-chain oligofructose causes a slow breakdown of fructans which leads to direct (stimulation of probiotics) and indirect (bone health, lipid metabolism, and prevents obstipation or diarrhea) effects in the colon. In addition to nutritional-health benefits, prebiotics (Synergy1) exhibits characteristic functional properties allowing its incorporation into a wide range of foods such as dairy, breads, and confectionaries [12]. 

Epidemiological studies in Asian populations demonstrate the influence of soybean consumption in the prevention of certain chronic diseases such as cancer and osteoporosis [13–15]. Soybean (Glycine max) is unique with phytochemicals such as isoflavones, saponins, phytates, protease inhibitors, phenolic acids, lecithin, dietary fiber, phytosterols, and omega-3-fatty acids. Metabolism of isoflavones such as genistin, daidzein and glycitin occurs in the presence of gut microflora that influences their bioavailability [16, 17].

Colonic adenomas are benign neoplastic polyps resulting from the accumulation of genetic alterations in normal colonic epithelium leading to malignant adenocarcinomas and metastasis [18–20]. Adenomas are useful biomarkers in evaluating the chemopreventive potential of various foods at different stages of cancer. Colons of F344 rats treated with AOM (potent colon-specific carcinogen) share similar histochemical properties to those of humans [21]. Therefore, AOM-F344 rat model is most extensively used in colon cancer research in identifying agents effective in control of the disease. Azoxymethane, due to its high potency, is usually administered as two injections with one week apart adequate dosage to induce colon cancer in rodents [21]. Although various studies have established the positive health benefits of prebiotics and soybean, it would be useful to understand the synergistic actions of these dietary ingredients at specific combinations that contribute as significant sources of fiber and protein in a normal balanced diet. The objective of the study was to evaluate the chemopreventive potential of prebiotics and soybean meal at 5% and 10% alone and in combinations in reducing colon cancer using a Fisher 344-rat model.

## 2. Materials and Methods

### 2.1. Animal Housing and Diets

**Figure 1:**
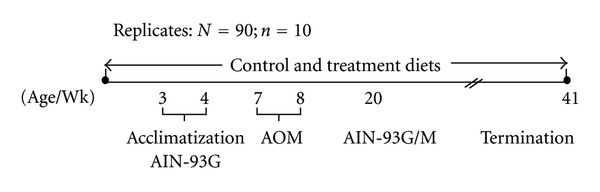
Experimental design of feeding control and treatment diets in F344 male rats. Scale is not proportional. (1) Control diet is based on AIN-93G/M (American Institute of Nutrition—93Growth/Maintenance) [22, 23]. (2) Treatment diets: 5%, 10% Prebiotics and Soybean meal fed singly and in combinations. (a) Rats: *N* = 90; *n* = 10 (Replicates = 5), (b) *T* ± 21°C; Relative humidity = 50%, (c) day and night = 12 hr. each, (d) Azoxymethane (AOM/colon specific carcinogen) dose = 16 mg/kg of body weight. The protocol involving animals was approved by the Institutional Animal Care and Use Committee of Alabama A&M University.

Ninety Fisher 344 male weanling rats (21 days old) were obtained from Harlan, Ind, USA, and housed in stainless steel wire cages at 2 rats per cage and acclimatized for one wk prior to administration of experimental diets. Experimental design is illustrated in Figure 1. Rats were randomly divided, assigned to nine groups (*n*  = 10), and fed the following diets: AIN-93G/M as control [22, 23] and treatment groups with prebiotics (5%), (10%), soybean meal (5%), (10%), prebiotics + soybean meal (5%  +  5%), (10%  +  10%), (5%  +  10%), and (10%  +  5%). Saline controls were used as negative controls in the study but not reported. Dietary modifications were made to fiber, casein and cornstarch (Table 1). All rats were housed and maintained according to standard protocol. Biweekly body weights and daily feed intakes were recorded. The diets were prepared once a month and stored at 4°C. Dietary ingredients were obtained from MP Biomedicals (Costa Mesa, Calif, USA). Prebiotics (Synergy1-Beneo) was obtained from Orafti (Teinen, Belgium), and soybean meal was obtained from a local natural food store (Garden Cove, Huntsville, Ala, USA), its composition is shown in Table 5. The protocol involving animals was approved by the Institutional Animal Care and Use Committee of Alabama A&M University.

### 2.2. Chemicals

All chemicals excluding Azoxymethane (Midwestern Research Institute, NCI, Chemical Repository, Kansas City, Mo, USA) were obtained from Sigma Chemical Company (St. Louis, Mo, USA).

### 2.3. Carcinogen Injection and Sample Collection

Colon tumors were induced by injecting rats with two s/c injections of azoxymethane (AOM) in saline at 16 mg/kg body wt. at 7 and 8 weeks of age. To validate the preventive role of test diets in colon cancer development, animals were injected with carcinogen after 3 week administration of the test diets. At 45 week of their age, all rats were killed using CO_2_ asphyxiation. Liver, colonic mucosal scrapings (CMS), and cecal samples were collected and stored at −80°C until further analysis. Femurs were harvested for mineral analysis.

### 2.4. Characterization of Colon Tumors

Tumor number, size, location, and TBR ratio (Tumors per tumor bearing rat ratio) were characterized [24].

### 2.5. Determination of Detoxification Enzyme

Glutathione-s-transferase (GST) activity (*μ*mol/mg) in the liver and CMS were assayed [25]. Absorbance was measured at 340 nm at the end of 5 minutes of reaction using a microplate reader (Synergy HT, Biotek, USA).

### 2.6. Determination of Antioxidative Enzyme

Hepatic catalase activity (*μ*mol/mg) was measured at 240 nm by monitoring the composition of H_2_O_2_ [26]. Total liver superoxide-dismutase (SOD) activity (*μ*mol/mg) was measured at 480 nm using xanthine oxidase as substrate [27].

### 2.7. Cecal Bacterial Enzyme Assays (*β*-Glucosidase and *β*-Glucuronidase)

Bacterial enzyme activity (*μ*mol/mL) of cecal contents was measured by the rate of p-nitrophenol release according to the modified method [28].

### 2.8. Bone Mineralization

Femurs were dry-ashed and prepared for analysis of selected minerals (Calcium-Ca, Phosphorus-P, Magnesium-Mg, Iron-Fe, and Zinc-Zn) in the bone using inductively coupled plasma (ICP) spectroscopy at specific wavelengths [29].

### 2.9. Statistical Analysis

Data were analyzed using SAS 9.1 statistical program (SAS, Cary, NC, USA). Results were expressed as means ± SEM. Significant differences among the treatment groups were determined by ANOVA, and means were separated using Tukey's studentized range test at *P*  ≤  0.05.

## 3. Results

### 3.1. Feed Intake, Weight Gain, Cecal Weight, and Cecal pH

There were no significant differences in feed intake (g/day) in rats fed control and treatment diets (Table 2). However, weight gain (g/41 wk) was significantly higher in rats fed prebiotics (5% and 10%) and SM (10%) compared to control. Rats fed combinational diets of prebiotics + SM (10% + 10% and 10% + 5%) had significantly lower weight gain compared to rats fed control and other treatment diets. An inverse relationship was observed between cecal weight and cecal pH in rats fed control and treatment diets (Table 3). Cecal weight (g) was lowest in control fed rats. Rats fed prebiotics (10%) singly and in combination with SM (10%), (10% + 5%) had significantly higher cecal weight (g) compared to other treatment fed rats. Among combination diet fed groups, prebiotics + SM (10% + 10% and 5% + 10%) had significantly lower cecal pH compared to other groups. However, rats fed prebiotics showed significantly higher cecal weight (g) and lower cecal pH among the rats fed singly. Cecal wall weight (g) ranged from 1.2 (control) to 3.8 (prebiotic-10%), and represents the absorbed residual fatty acids in the wall of cecum.

### 3.2. Distribution and Characterization of Colonic Tumors

#### 3.2.1. Tumor Incidence

**Figure 2:**
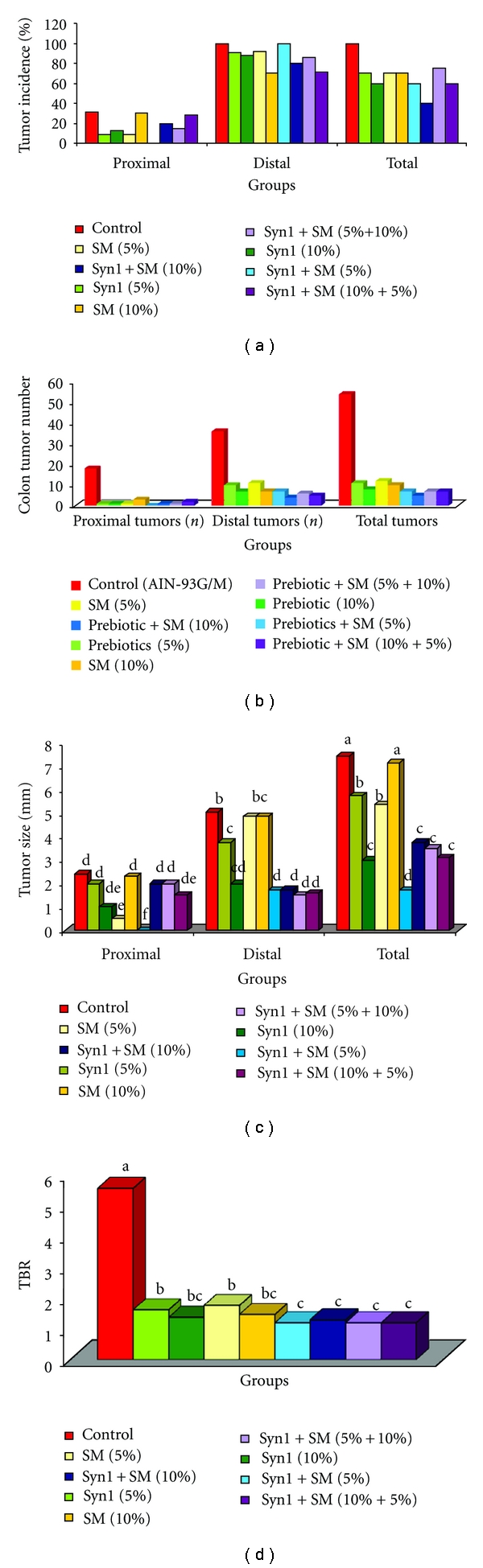
(a) Effect of feeding prebiotics and soybean meal on colon tumor incidence (percentage) in F344 male rats. (b) Effect of feeding prebiotics and soybean meal on colon tumor number (*n*) in F344 male rats. (c) Effect of feeding prebiotics and soybean meal on colonic tumor size (mm) in F344 male rats. Values are expressed as means ± SEM. ^abcdef^Bars with same letter are not significantly different using Tukey's studentized range test (*P* ≤ 0.05). (d) Effect of feeding prebiotics and soybean meal on tumors per tumor bearing rat ratio (TBR) in F344 male rats. *N* ^1^ represents the number of rats with tumors; *N* ^2^ is total number of rats at the end of the experiment. Values are expressed as means ± SEM. ^abc^Bars with same letter are not significantly different using Tukey's studentized range test (*P* ≤ 0.05).

The percentage tumor incidence in rats fed control and treatment diets were higher in the distal colon compared to the proximal (Figure 2(a)). Rats fed control diet had higher tumor induction in proximal and distal colons compared to the rats fed treatment diets. Among the treatment groups, reductions in tumor incidence (%) in rats fed prebiotics and SM ranged from 25 to 40 compared to C. However, rats fed combinations of prebiotics and SM (10%) had the lowest tumor incidence (40%).

#### 3.2.2. Tumor Number

Rats fed control diets had highest tumor numbers in both proximal (18) and distal colon (36). Reductions (%) in total tumors in rats fed treatment diets ranged from a low of 77.7 (SM-5%) to high of 90.7 (prebiotics + SM-10%) compared to C (Figure 2(b)). Among rats fed treatment diets, prebiotics (10%) and combination diet fed rats (prebiotics + SM-10%) had the lowest number of total tumors. No proximal tumors were seen in rats fed (prebiotics  +  SM-5%).

#### 3.2.3. Tumor Size

Compared to control fed rats, rats fed treatment diets had smaller tumor (mm) both in the proximal and distal colon (Figure 2(c)). Rats fed control diet, prebiotics, and SM singly had larger tumor (mm) in distal than proximal colon. However, rats fed combination diets of prebiotics + SM (10%, 5% + 10%) had smaller tumor (mm) in distal colon. Reductions (%) in tumor size (mm) in rats fed combination diets of prebiotics and SM ranged from a low of 50 (prebiotics + SM-10%) to high of 77.7 (prebiotics  + SM-5%).

#### 3.2.4. Tumors/Tumor-Bearing Rat Ratio (TBR)

Rats fed the control diet had higher (5.4) tumors/tumor-bearing rat (TBR) ratio (Figure 2(d)). TBR in rats fed treatment diets ranged from 1.16 to 1.71. TBR ratios were similar in rats fed combination diets except in rats fed prebiotics + SM (10%). Reductions (%) in TBR ratio in rats fed single treatment diets ranged from a low of 62.2 (SM-5%) to high of 71.1 (prebiotics 10%) and in rats fed combination diets ranged from 73.3 (prebiotics + SM-10%) to 74.2 (5%, 5% + 10% and 10% + 5%) compared to control. Overall, rats fed combination diets had reduced TBR, tumor number, and smaller tumor (mm) compared to rats fed prebiotics and SM singly (Table 5).

### 3.3. Hepatic and Colonic Glutathione-s-Transferase (GST) Activities

**Figure 3:**
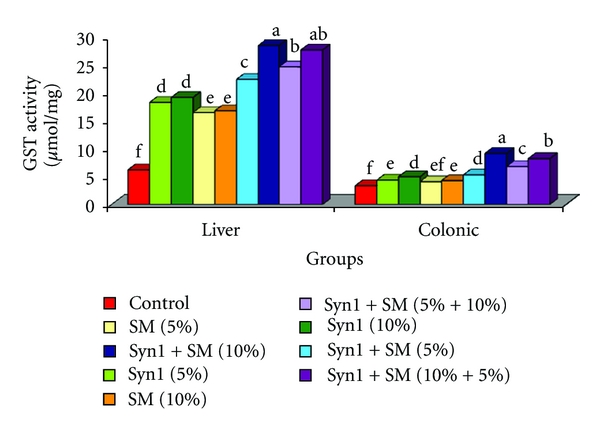
Effect of feeding prebiotics and soybean meal on (a) hepatic GST (b) colonic GST activity (*μ*mol/mg) in F344 male rats. Abbreviations: SM: soybean meal, GST: glutathione-s-transferase. Values are expressed as means ± SEM. ^abcdef^Bars with same letter are not significantly different using Tukey's studentized range test (*P* ≤ 0.05).

Liver GST activity (*μ*mol/mg) in rats fed treatment diets was significantly higher than control fed rats (Figure 3(a)). GST activity (*μ*mol/mg) in treatment groups ranged from a low of 16.4 (SM-5%) to high of 28.3 (prebiotics + SM-10%). There was over two- to fourfold increase in hepatic GST activity (*μ*mol/mg) in rats fed treatment diets compared to the control fed rats. Among treatment groups, rats fed combination diets of prebiotics + SM showed significantly higher GST activity (*μ*mol/mg) than rats fed SM singly. Similar trends were observed with CMS GST activities (*μ*mol/mg) (Figure 3(b)). CMS GST activities (*μ*mol/mg) were significantly higher in rats fed SM (10%), prebiotics + SM (5%, 10%, 5% + 10%, 10% + 5%) compared to control fed rats. Among rats fed combination diets, colonic GST activity (*μ*mol/mg) ranged from a low of 5.2 (prebiotics + SM-5%) to high of 9.0 (prebiotics + SM).

### 3.4. Antioxidative Enzyme Activities

**Figure 4:**
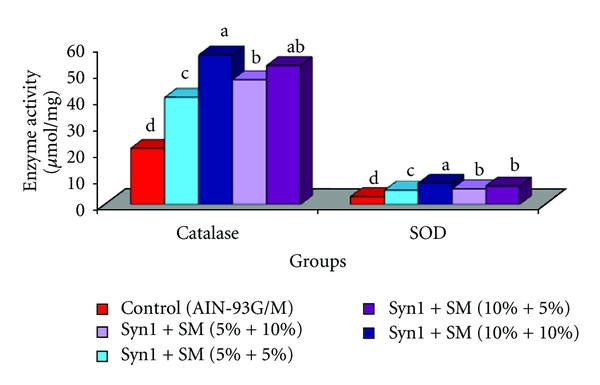
Effect of feeding prebiotics and soybean meal on hepatic antioxidative enzyme activities (a) Catalase (b) SOD activity (*μ*mol/mg) in F344 male rats. Abbreviations: SM: soybean meal, SOD: Superoxide-dismutase. ^abcd^Bars with same letter are not significantly different using Tukey's studentized range test (*P* ≤ 0.05).

Catalase activity (CAT) was significantly higher in rats fed prebiotic and SM in combinations compared to the control rats (Figure 4(a)). Among treatment groups, rats fed prebiotic  +  SM (10%) had highest (56.3) catalase activity (*μ*mol/mg), accounting for a two fold increase in rats fed treatment diets. Rats fed control diet showed significantly lower superoxide dismutase activity (SOD) (*μ*mol/mg) compared to rats fed treatment diets (Figure 4(b)). SOD activity (*μ*mol/mg) ranged from a low of 2.9 ± 0.09 in rats fed the control diet to a high of 8.0 ± 0.11 in rats fed prebiotic + SM (10%). CAT and SOD activities (*μ*mol/mg) were two–four folds higher in rats fed combination diets compared to control.

### 3.5. Cecal Bacterial Enzyme Activities

**Figure 5:**
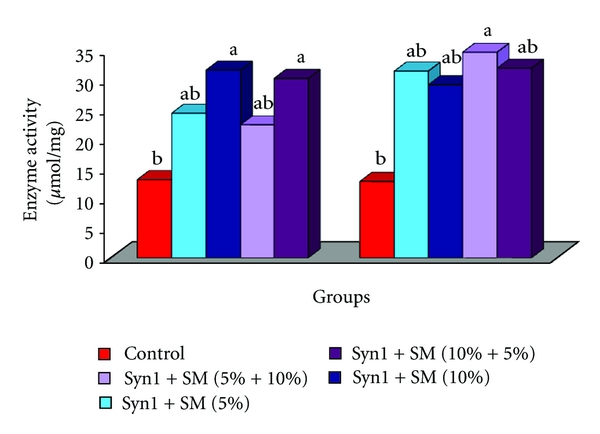
Effect of feeding prebiotics and soybean meal on cecal bacterial enzyme activities. (a) *β*-glucosidase. (b) *β*-glucuronidase activity (*μ*mol/mL) in F344 male rats. Abbreviations: SM: soybean meal. ^ab^Bars with same letter are not significantly different using Tukey's studentized range test (*P* ≤ 0.05).

Rats fed prebiotics + SM (10%, 10% + 5%) had significantly higher cecal *β*-glucosidase activity (*μ*mol/mL) compared to control (Figure 5(a)). However, no significant differences were observed in cecal *β*-glucosidase activity (*μ*mol/mL) between the rats fed SM singly and prebiotics + SM (5%, 5% + 10%) and to control fed rats. Cecal *β*-glucuronidase activity (*μ*mol/mL) was significantly higher in rats fed SM (10%) singly and prebiotics + SM (5% + 10%) compared to control (Figure 5(b)). Cecal *β*-glucuronidase activity (*μ*mol/mL) ranged from a low of 28.9 (prebiotics + SM-10%) to high of 34.3 (prebiotics + SM (5% + 10%)).

### 3.6. Bone Mineralization

Minerals measured in femurs were calcium (Ca), phosphorus (P), magnesium (Mg), iron (Fe), and zinc (Zn) (Table 4). Ca (mg/g) was significantly higher in rats fed SM (10%) singly and in combination with prebiotics than rats fed control diets. Among rats fed combination diets, prebiotics + SM (10%) group had the highest bone calcium (mg/g). Phosphorus (mg/g) was significantly lower in rats fed control diet compared to treatment fed rats. Increase (%) in bone phosphorus (mg/g) was highest (42.6) in rats fed prebiotics + SM (10% + 5%). Bone Mg (mg/g) was significantly higher in rats fed treatment diets compared to control (2.2). Among treatment fed rats, the group fed prebiotics + SM (10%) had highest bone Mg (mg/g) (6). Bone Fe and Zn (*μ*g/g) were significantly lower in rats fed control diet compared to treatment fed rats (Table 4). Although no significant differences were seen in bone Fe (*μ*g/g) among the treatment groups, there was over twofold increase in bone Fe (*μ*g/g) compared to control fed rats (53.1). Bone Zn (*μ*g/g) among treatment fed rats ranged from 530 (SM-5%) to 741 (prebiotics + SM-10%).

## 4. Discussion

Consumption of a balanced diet rich in various phytochemicals may provide primary prevention against chronic diseases. This study evaluated the combinational effects of prebiotics and soybean in prevention of colon carcinogenesis. Although no significant differences were observed in feed intake (g/rat/day) among control and treatment groups, the average body weights of rats at the end of the experiment (41 wk) ranged from 300–400 g. Rats fed treatment diets in combination had lower weight gain (g/41 wk) compared to rats fed the control and treatment diets singly. Combined effects of prebiotics and SM in decreasing weight gain may be explained by the influence of short chain fatty acids (propionate) produced by colonic fermentation exerting hypolipidemic effects through decreased lipogenesis in liver, thereby reduced concentration of plasma very low-density lipoproteins (VLDL) [30–32]. Similar trend was reported studying the inhibitory effects of different inulin fractions in Fisher 344 male rats [33]. Cecal fermentation of soluble dietary fiber (prebiotics) by intestinal microflora is well documented [34–36]. Cecal weight and cecal pH showed an inverse relationship in rats fed prebiotics singly and in specific combinations (prebiotics + SM-10% + 10%; 10% + 5%). Reduction in cecal pH is critical for balanced colonic microflora to support colon physiology, prevention of colonic diseases, and in metabolism of phytonutrients such as isoflavones [37]. Increased cecal weight from prebiotics consumption may result in short chain fatty acids (SCFA) promoting cecal growth as observed *in vivo* using inulin in various studies [28, 38], where a positive correlation between a lower cecal pH and colon tumor reductions was also seen in our study.

In the current study, we observed a decrease in tumor incidence (40%–70%) as well as tumor size (mm), tumor number, and TBR in both proximal and distal sections in rats fed treatments diets in combinations compared to the control. Tumor number and tumor size are indicators of proliferation and angiogenesis/inflammation, while TBR represents tumor multiplicity. Similar results were seen in rats fed 10% inulin [5]. Changes in tumor growth characteristics observed in rats fed combination diets suggests antiproliferative, antiangiogenic and overlapping actions of prebiotics and soybean meal. Indirect defensive mechanisms of phytochemicals involve either stimulation or inhibition of crucial detoxification and antioxidative enzymes [39, 40]. Detoxification of xenobiotics in the liver is a primary strategy of the biological system in cancer prevention. Stimulation of hepatic and colonic glutathione-s-transferase (GST) activity (*μ*mol/mg) in rats fed combination diets is indicative of the protective effects of prebiotics and SM in stimulation of the enzyme. Colonic GST activity provides residual detoxification effects in xenobiotic metabolism. Our results are in agreement with similar studies, where GST activity was significantly induced when Fisher 344 male rats were fed with Flax seed meal at 10%, 20% and silymarin at 100, 500, and 1000 ppm [41, 42]. Antioxidative enzymes in liver such as catalase and superoxide-dismutase (SOD) were stimulated in rats fed treatment diets with highest activities seen in rats fed combination diets. Stimulation of antioxidative enzymes by phytochemicals present in plant foods such as soybean may be attributed to the structure of polyphenols (OH groups) and their metabolites such as equol which has enhanced antioxidative potential [43–45]. Various studies support the stimulation of antioxidants by phytochemicals as one of their protective mechanisms in the prevention of chronic diseases such as cancer [46–48]. Physiologically, induction of detoxifying and antioxidative enzymes by dietary bioactive compounds such as soluble fiber (prebiotics) and isoflavones (soybean), their byproducts, and metabolites, may contribute to the cellular defensive mechanisms [49–51].

Cecal microflora and their enzyme activities play a prominent role in the pathology of colonic disease. Establishment and modulation of colonic microflora is largely influenced by diet [52, 53]. *β*-glucosidase is a gut microbial enzyme catalyzing the hydrolysis of isoflavone glycan conjugates to aglycans, thus enhancing their bioavailability, while *β*-glucuronidase are enzymes involved in deconjugation of glycosylated, sulfated, and glucuronidated forms of metabolites regulated by biliary secretions [54]. In our study, *β*-glucosidase and *β*-glucuronidase (*μ*mol/mL) were higher in rats fed treatment diets. Our results were in agreement with a study involving Fisher 344 rats fed fructo-oligosaccharides (Raftilose P95) [55]. However, results on cecal *β*-glucosidase and *β*-glucuronidase activities are conflicting in rats fed Inulin and sucrose at 5% levels [56]. Experimental studies in animals and humans have shown positive effects of ingesting synbiotics as well as soybean isoflavones on mineral absorption, bone structure, and health [57]. Underlying mechanisms of calcium absorption in the presence of intestinal fermentation and isoflavone metabolites contributing to a balanced bone remodeling have been illustrated [37]. In the present study, rats fed combination diets of prebiotics and SM showed higher bone mineralization compared to rats fed control and SM singly. Our results corroborate previous studies [58, 59], which showed the effects of fructo-oligosaccharides, isoflavones, and their metabolites in maintaining bone health.

## 5. Conclusions

Results indicate a pronounced chemopreventive effect of prebiotics and soybean in combinations rather than when fed singly. Reductions in tumor incidence, smaller tumor size (mm), and lower tumor number may have been attributed to the direct effects of treatment diets by acting as antiproliferative and antiangiogenic factors or by indirect mechanism such as stimulation of detoxifying and antioxidative enzymes. Interactive mechanisms of prebiotics and soybean may have contributed to tumor reductions. Prebiotics has been associated in the prevention of gut-associated disorders and in isoflavone metabolism. Metabolites of soybean isoflavones such as equol and des-methylangolensin may play a role in enhancing the chemoprotective role of prebiotics in colon cancer. Further, exploring the synergistic effects of phytonutrients and their metabolites on microbial enzymatic activities associated with gut, on cellular and molecular targets such as specific genes with implications in cancer prevention, may be promising.

## Figures and Tables

**Table 1:** Composition of diets^a^ (AIN93-M).

Ingredients	Control AIN93M	Prebiotic 5%	Prebiotic 10%	SM 5%	SM 10%	Prebiotic 5% + SM 5%	Prebiotic 10% + SM 10%	Prebiotic 5% + SM 10*% *	Prebiotic 10% + SM 5%
Corn starch	465.7	415.7	365.7	439.7	413.7	389.7	313.7	363.7	339.7
Casein	140	140	140	120	100	120	100	100	120
Fiber	50	50	50	46	42	46	42	42	46
Prebiotic	0	50	100	0	0	50	100	50	100
SM	0	0	0	50	100	50	100	100	50
Common^b^ Ingredients	344.3	344.3	344.3	344.3	344.3	344.3	344.3	344.3	344.3

^a^Formulations of diets based on AIN-93M [22, 23].

^b^Common ingredients (g): dextrose, 155; sucrose, 100; soybean oil, 40 g; mineral mix (AIN-93M), 35; vitamin mix, 10; L-cysteine, 1.8; choline bitatrate, 2.5.

Abbreviations: SM: soybean meal.

**Table 2:** Feed intake and weight gain in rats fed prebiotic and soybean meal.

Groups	Feed intake (g/day)	Weight gain (g/41 wk)
Control (AIN-93G/M)	17.6 ± 0.91	310.2 ± 8.3^b^
Prebiotic** (**5%)	18.04 ± 0.6	361.4 ± 5.8^a^
Prebiotic (10%)	18.92 ± 0.6	370.1 ± 8.98^a^
SM (5%)	17.2 ± 0.4	353.3 ± 6.3^ab^
SM (10%)	18.2 ± 0.3	358.5 ± 8.2^a^
Prebiotic + SM (5% + 5%)	17.8 ± 0.5	327.0 ± 7.8^b^
Prebiotic + SM (10% + 10%)	18.1 ± 0.6	285.5 ± 6.3^c^
Prebiotic + SM (5% + 10%)	17.2 ± 0.4	333.5 ± 6.0^ab^
Prebiotic + SM (10% + 5%)	17.7 ± 0.4	286.6 ± 6.1^c^

Abbreviations: SM: soybean meal, values are expressed as means ± SEM.

^abc^Means in a column with the same letter are not significantly different using Tukey's studentized range test (*P*  ≤  0.05).

**Table 3:** Effect of prebiotics and soybean meal on cecal weight and cecal pH.

Groups	Total cecal weight (g)	Cecal wall weight (g)	Cecal pH
Control (AIN-93G/M)	3.7 ± 0.22^c^	1.2 ± 0.1^b^	7.82 ± 0.03^a^
Prebiotic (5%)	5.0 ± 0.3^b^	2.9 ± 0.2^a^	6.4 ± 0.05^e^
Prebiotic (10%)	7.1 ± 0.4^a^	3.8 ± 0.3^a^	6.2 ± 0.10^e^
SM (5%)	3.9 ± 0.3^bc^	2.2 ± 0.2^ab^	7.6 ± 0.05^a^
SM (10%)	4.3 ± 0.2^bc^	2.2 ± 0.1^ab^	7.7 ± 0.04^a^
Prebiotic + SM (5% + 5%)	5.1 ± 0.2^b^	1.9 ± 0.1^b^	7.3 ± 0.06^b^
Prebiotic + SM (10% + 10%)	7.2 ± 0.4^a^	3.0 ± 0.1^a^	6.7 ± 0.03^d^
Prebiotic + SM (5% + 10%)	5.4 ± 0.5^b^	2.4 ± 0.2^ab^	6.9 ± 0.08^c^
Prebiotic + SM (10% + 5%)	6.8 ± 0.3^a^	2.5 ± 0.2^ab^	6.7 ± 0.04^cd^

Abbreviations: SM: soybean meal.

Values are expressed as means ± SEM.

^abcde^Means in a column with the same letter are not significantly different using Tukey's studentized range test (*P*  ≤  0.05).

**Table 4:** Effect of prebiotic and soybean meal on bone health.

Groups	Ca (mg/g)	P (mg/g)	Mg (mg/g)	Fe (*μ*g/g)	Zn (*μ*g/g)
Control	267.5 ± 11.2^d^	122.4 ± 1.2^d^	2.2 ± 0.1^c^	53.1 ± 0.8^b^	163.9 ± 24.8^f^
Prebiotic (5%)	276.1 ± 8.4^c^	146.2 ± 2.6^bc^	3.6 ± 0.2^b^	96.2 ± 2.9^a^	502.1 ± 6.8^e^
Prebiotic (10%)	282.6 ± 11.1^c^	152.9 ± 4.8^c^	4.0 ± 0.9^b^	104.1 ± 4.2^a^	528.6 ± 6.2^d^
SM (5%)	268.5 ± 26.9^d^	133.6 ± 1.8^c^	3.4 ± 0.17^b^	108.3 ± 5.3^a^	530.9 ± 9.3^d^
SM (10%)	277.8 ± 36.5^c^	141.0 ± 3.4^bc^	3.8 ± 0.06^b^	105.6 ± 1.4^a^	574.5 ± 34.6^c^
Prebiotic + SM (5% + 5%)	288.9 ± 5.8^c^	159.3 ± 4.4^b^	4.1 ± 0.13^b^	119.1 ± 0.8^a^	702.8 ± 29.3^ab^
Prebiotic + SM (10% + 10%)	**431.4 ± 3.3** ^**a**^	167.8 ± 2.8^ab^	**6.0 ± 0.7** ^**a**^	114.7 ± 5.8^a^	**714.3 ± 35.0** ^**a**^
Prebiotic + SM (5% + 10%)	329.2 ± 14.7^bc^	166.4 ± 10.9^ab^	4.5 ± 0.41^ab^	112.8 ± 8.4^a^	**741.0 ± 34.6** ^**a**^
Prebiotic+SM (10% + 5%)	395.3 ± 1.1^b^	**174.0 ± 6.9** ^**a**^	4.1 ± 0.4^b^	114.9 ± 6.0^a^	654.4 ± 22.**7** ^**b**^

Abbreviations: SM: soybean meal, Ca: Calcium, P: Phosphorus, Mg: Magnesium, Fe: Iron, Zn: Zinc.

Values are expressed as means ± SEM.

^abcdef^Means in a column with the same letter are not significantly different using Tukey's studentized range test (*P*  ≤  0.05).

**Table 5:** Composition of defatted whole dry soybean meal (low fat).

Serving size	19 g
Calories	80.00
Calories from fat	15.00
Total fat	1.50 g
Saturated fat	0.00 g
Trans fat	0.00 g
Cholesterol	0.00 mg
Sodium	0.00 mg
Total carbohydrate	5.00 g
Dietary fiber	3.00 g
Sugars	2.00 g
Protein	10.00 g

## References

[1] ACS, American Cancer Society Facts, Figures and Statistics. http://www.acs.org.

[2] Yabroff KR, Mariotto AB, Feuer E, Brown ML (2008). Projections of the costs associated with colorectal cancer care in the United States, 2000–2020. *Health Economics*.

[3] Steinmetz KA, Potter JD (1996). Vegetables, fruit, and cancer prevention: a review. *Journal of the American Dietetic Association*.

[4] Potter JD (1993). Colon cancer—do the nutritional epidemiology, the gut physiology and the molecular biology tell the same story?. *Journal of Nutrition*.

[5] Verghese M, Rao DR, Chawan CB, Williams LL, Shackelford L (2002). Dietary inulin suppresses azoxymethane-induced aberrant crypt foci and colon tumors at the promotion stage in young Fisher 344 rats. *Journal of Nutrition*.

[6] Agurs-Collins T, Smoot D, Afful J, Makambi K, Adams-Campbell LL (2006). Legume intake and reduced colorectal adenoma risk in African-Americans. *Journal of National Black Nurses’ Association*.

[7] Boateng J, Verghese M, Chawan CB (2006). Red palm oil suppresses the formation of azoxymethane (AOM) induced aberrant crypt foci (ACF) in Fisher 344 male rats. *Food and Chemical Toxicology*.

[8] O’Keefe SJD, Chung D, Mahmoud N (2007). Why do African Americans get more colon cancer than Native Africans?. *Journal of Nutrition*.

[9] Femia AM, Caderni G (2008). Rodent models of colon carcinogenesis for the study of chemopreventive activity of natural products. *Planta Medica*.

[10] De Kok TM, Van Breda SG, Manson MM (2008). Mechanisms of combined action of different chemopreventive dietary compounds: a review. *European Journal of Nutrition*.

[11] Lim CC, Ferguson LR, Tannock GW (2005). Dietary fibres as “prebiotics”: implications for colorectal cancer. *Molecular Nutrition and Food Research*.

[12] Kolida S, Tuohy K, Gibson GR (2002). Prebiotic effects of inulin and oligofructose. *British Journal of Nutrition*.

[13] Toyomura K, Kono S (2002). Soybeans, soy foods, isoflavones and risk of colorectal cancer: a review of experimental and epidemiological data. *Asian Pacific Journal of Cancer Prevention*.

[14] Messina MJ, Persky V, Setchell KDR, Barnes S (1994). Soy intake and cancer risk: a review of the in vitro and in vivo data. *Nutrition and Cancer*.

[15] Messina MJ (1999). Legumes and soybeans: overview of their nutritional profiles and health effects. *American Journal of Clinical Nutrition*.

[16] Larkin T, Price WE, Astheimer L (2008). The key importance of soy isoflavone bioavailability to understanding health benefits. *Critical Reviews in Food Science and Nutrition*.

[17] Raimondi S, Roncaglia L, De Lucia M (2009). Bioconversion of soy isoflavones daidzin and daidzein by Bifidobacterium strains. *Applied Microbiology and Biotechnology*.

[18] Aksoy N, Akinci OF (2004). Mucin macromolecules in normal, adenomatous, and carcinomatous colon: evidence for the neotransformation. *Macromolecular Bioscience*.

[19] Krishnan K, Ruffin MT, Brenner DE (2000). Chemoprevention for colorectal cancer. *Critical Reviews in Oncology/Hematology*.

[20] Srivastava S, Verma M, Henson DE (2001). Biomarkers for early detection of colon cancer. *Clinical Cancer Research*.

[21] Reddy BS (2004). Studies with the azoxymethane-rat preclinical model for assessing colon tumor development and chemoprevention. *Environmental and Molecular Mutagenesis*.

[22] Reeves PG, Nielsen FH, Fahey GC (1993). AIN-93 purified diets for laboratory rodents: final report of the American Institute of Nutrition ad hoc writing committee on the reformulation of the AIN-76A rodent diet. *Journal of Nutrition*.

[23] Reeves PG, Rossow KL, Lindlauf J (1993). Development and testing of the AIN-93 purified diets for rodents: results on growth, kidney calcification and bone mineralization in rats and mice. *Journal of Nutrition*.

[24] Shackelford LA, Ramkishan Rao D, Chawan CB, Pulusani SR (1983). Effect of feeding fermented milk on the incidence of chemically induced colon tumors in rats. *Nutrition and Cancer*.

[25] Habig WH, Pabst MJ, Jakoby WB (1974). Glutathione S transferases. The first enzymatic step in mercapturic acid formation. *Journal of Biological Chemistry*.

[26] Aebi H (1984). Catalase in vitro. *Methods in Enzymology*.

[27] Fridovich I (1989). Superoxide dismutases. An adaptation to a paramagnetic gas. *Journal of Biological Chemistry*.

[28] Zduńczyk Z, Juśkiewicz J, Estrella I (2006). Cecal parameters of rats fed diets containing grapefruit polyphenols and inulin as single supplements or in a combination. *Nutrition*.

[30] Delzenne NM, Cani PD, Neyrinck AM (2007). Modulation of glucagon-like peptide 1 and energy metabolism by inulin and oligofructose: Experimental data. *Journal of Nutrition*.

[31] Ørgaard A, Jensen L (2008). The effects of soy isoflavones on obesity. *Experimental Biology and Medicine*.

[32] Cani PD, Dewever C, Delzenne NM (2004). Inulin-type fructans modulate gastrointestinal peptides involved in appetite regulation (glucagon-like peptide-1 and ghrelin) in rats. *British Journal of Nutrition*.

[33] Verghese M, Walker LT, Shackelford L, Chawan CB (2005). Inhibitory effects of nondigestible carbohydrates of different chain lengths on azoxymethane-induced aberrant crypt foci in Fisher 344 rats. *Nutrition Research*.

[34] Pirman T, Ribeyre MC, Mosoni L (2007). Dietary pectin stimulates protein metabolism in the digestive tract. *Nutrition*.

[35] Alabaster O, Tang Z, Shivapurkar N (1996). Dietary fiber and the chemopreventive modelation of colon carcinogenesis. *Mutation Research*.

[36] Cummings JH, Macfarlane GT, Englyst HN (2001). Prebiotic digestion and fermentation. *American Journal of Clinical Nutrition*.

[37] Coxam V (2007). Current data with inulin-type fructans and calcium, targeting bone health in adults. *Journal of Nutrition*.

[38] Levrat MA, Remesy C, Demigne C (1991). High propionic acid fermentations and mineral accumulation in the cecum of rats adapted to different levels of inulin. *Journal of Nutrition*.

[39] Wilkinson J, Clapper ML (1997). Detoxication enzymes and chemoprevention. *Proceedings of the Society for Experimental Biology and Medicine*.

[40] Clapper ML, Szarka CE (1998). Glutathione S-transferases-biomarkers of cancer risk and chemopreventive response. *Chemico-Biological Interactions*.

[41] Williams D, Verghese M, Walker LT, Boateng J, Shackelford L, Chawan CB (2007). Flax seed oil and flax seed meal reduce the formation of aberrant crypt foci (ACF) in azoxymethane-induced colon cancer in Fisher 344 male rats. *Food and Chemical Toxicology*.

[42] Kohno H, Tanaka T, Kawabata K (2002). Silymarin, a naturally occurring polyphenolic antioxidant flavonoid, inhibits azoxymethane-induced colon carcinogenesis in male F344 rats. *International Journal of Cancer*.

[43] Kampkotter A, Chovolou Y, Kulawik A (2008). Isoflavone daidzein possesses no antioxidant activities in cell-free assays but induces the antioxidant enzyme catalase. *Nutrition Research*.

[44] Yuan JP, Wang JH, Liu X (2007). Metabolism of dietary soy isoflavones to equol by human intestinal microflora—implications for health. *Molecular Nutrition and Food Research*.

[45] Mitchell JH, Gardner PT, McPhail DB, Morrice PC, Collins AR, Duthie GG (1998). Antioxidant efficacy of phytoestrogens in chemical and biological model systems. *Archives of Biochemistry and Biophysics*.

[46] Manju V, Nalini N (2005). Chemopreventive efficacy of ginger, a naturally occurring anticarcinogen during the initiation, post-initiation stages of 1,2 dimethylhydrazine-induced colon cancer. *Clinica Chimica Acta*.

[47] Stoner GD, Mukhtar H (1995). Polyphenols as cancer chemopreventive agents. *Journal of Cellular Biochemistry*.

[48] Aranganathan S, Panneer Selvam J, Nalini N (2009). Hesperetin exerts dose dependent chemopreventive effect against 1,2-dimethyl hydrazine induced rat colon carcinogenesis. *Investigational New Drugs*.

[49] Sauer J, Richter KK, Pool-Zobel BL (2007). Products formed during fermentation of the prebiotic inulin with human gut flora enhance expression of biotransformation genes in human primary colon cells. *British Journal of Nutrition*.

[50] Sauer J, Richter KK, Pool-Zobel BL (2007). Physiological concentrations of butyrate favorably modulate genes of oxidative and metabolic stress in primary human colon cells. *Journal of Nutritional Biochemistry*.

[51] Rimbach G, De Pascual-Teresa S, Ewins BA (2003). Antioxidant and free radical scavenging activity of isoflavone metabolites. *Xenobiotica*.

[52] Gorbach SL (1982). The intestinal microflora and its colon cancer connection. *Infection*.

[53] McGarr SE, Ridlon JM, Hylemon PB (2005). Diet, anaerobic bacterial metabolism, and colon cancer: a review of the literature. *Journal of Clinical Gastroenterology*.

[54] Roberton AM, Lee SP, Lindop R, Stanley RA, Thomsen L, Tasman-Jones C (1982). Biliary control of beta-glucuronidase activity in the luminal contents of the rat ileum, cecum, and rectum. *Cancer Research*.

[55] Djouzi Z, Andrieux C (1997). Compared effects of three oligosaccharides on metabolism of intestinal microflora in rats inoculated with a human faecal flora. *British Journal of Nutrition*.

[56] Juskiewicz J, Zdunczyk Z, Frejnagel S (2007). Caecal parameters of rats fed diets supplemented with inulin in exchange for sucrose. *Archives of Animal Nutrition*.

[57] Scholz-Ahrens KE, Ade P, Marten B (2007). Prebiotics, probiotics, and synbiotics affect mineral absorption, bone mineral content, and bone structure. *Journal of Nutrition*.

[58] Mathey J, Puel C, Kati-Coulibaly S (2004). Fructooligosaccharides maximize bone-sparing effects of soy isoflavone-enriched diet in the ovariectomized rat. *Calcified Tissue International*.

[59] Ohta A, Uehara M, Sakai K (2002). A combination of dietary fructooligosaccharides and isoflavone conjugates increases femoral bone mineral density and equol production in ovariectomized mice. *Journal of Nutrition*.

